# Lack of synergistic effect of resveratrol and sigma-1 receptor agonist (PRE-084) in SOD1^G93A^ ALS mice: overlapping effects or limited therapeutic opportunity?

**DOI:** 10.1186/1750-1172-9-78

**Published:** 2014-05-21

**Authors:** Renzo Mancuso, Jaume del Valle, Marta Morell, Mercé Pallás, Rosario Osta, Xavier Navarro

**Affiliations:** 1 Institute of Neurosciences and Department of Cell Biology, Physiology and Immunology, Universitat Autònoma de Barcelona, and Centro de Investigación Biomédica en Red sobre Enfermedades Neurodegenerativas (CIBERNED), Bellaterra, Spain; 2 Unitat de Farmacologia i Farmacognòsia, Facultat de Farmàcia, Institut de Biomedicina (IBUB), Universitat de Barcelona, and CIBERNED, Barcelona, Spain; 3 Laboratory of Genetic Biochemistry (LAGENBIO-I3A), Aragon Institute of Health Sciences, Universidad de Zaragoza, Zaragoza, Spain; 4 Unitat de Fisiologia Mèdica, Facultat de Medicina, Universitat Autònoma de Barcelona, Bellaterra E-08193, Spain

**Keywords:** Motoneuron disease, Amyotrophic lateral sclerosis, Resveratrol, Sirtuin 1, AMPK, SOD1^G93A^ mice, Sigma-1 receptor, PRE-084, Combined therapy

## Abstract

**Background:**

Amyotrophic lateral sclerosis (ALS) is an adult onset neurodegenerative disease characterized by the loss of motoneurons (MNs) in the spinal cord, brainstem and motor cortex, causing progressive paralysis and death. Nowadays, there is no effective therapy and most patients die 2–5 years after diagnosis. Sigma-1R is a transmembrane protein highly expressed in the CNS and specially enriched in MNs. Mutations on the Sigma-1R leading to frontotemporal lobar degeneration-ALS were recently described in human patients. We previously reported the therapeutic role of the selective sigma-1R agonist 2-(4-morpholi-nethyl)1-phenylcyclohexanecarboxylate (PRE-084) in SOD1^G93A^ ALS mice, that promoted spinal MN preservation and extended animal survival by controlling NMDA receptor calcium influx. Resveratrol (RSV, trans-3,4′,5-trihydroxystilbene) is a natural polyphenol with promising neuroprotective effects. We recently found that RSV administration to SOD1^G93A^ mice preserves spinal MN function and increases mice survival. These beneficial effects were associated to activation of Sirtuin 1 (Sirt1) and AMP-activated protein kinase (AMPK) pathways, leading to the modulation of autophagy and an increase of mitochondrial biogenesis. The main goal of this work was to assess the effect of combined RSV and PRE-084 administration in SOD1^G93A^ ALS mice.

**Methods:**

We determined the locomotor performance of the animals by rotarod test and evaluated spinal motoneuron function using electrophysiological tests.

**Results:**

RSV plus PRE-084 treatment from 8 weeks of age significantly improved locomotor performance and spinal MN function, accompanied by a significant reduction of MN degeneration and an extension of mice lifespan. In agreement with our previous findings, there was an induction of PKC-specific phosphorylation of the NMDA-NR1 subunit and an increased expression and activation of Sirt1 and AMPK in the ventral spinal cord of treated SOD1^G93A^ animals.

**Conclusions:**

Although combined PRE and RSV treatment significantly ameliorated SOD1^G93A^ mice, it did not show a synergistic effect compared to RSV-only and PRE-084-only treated groups.

## Introduction

Amyotrophic lateral sclerosis (ALS) is an adult onset neurodegenerative disease characterized by the loss of motoneurons (MN) in the spinal cord, brainstem and motor cortex. It clinically manifests by progressive weakness, muscle atrophy and paralysis [1,2]. The majority of ALS cases are sporadic with unknown etiology but 10% of them are inherited forms, linked to genetic mutations. Mutations in the gene encoding for the enzyme Cu/Zn superoxide dismutase 1 (SOD1) are observed in about 20% of the familial cases of ALS [3]. The transgenic mouse that over-expresses the human mutated form of the SOD1 gene with a glycine to alanine conversion at the 93rd codon is the most studied model [4-6] and recapitulates the main features of both sporadic and familial ALS forms [5]. SOD1 protein alterations have also been reported in sporadic ALS patients [7].

The sigma-1 receptor (sigma-1R) is an endoplasmic reticulum (ER) transmembrane protein [8] highly expressed in spinal MNs [9-11], and specially enriched in postsynaptic sites of C-terminals [12]. Sigma-1R participates in several cellular processes including neuritogenesis, ionic channels conductance, calcium homeostasis and microglial activity [13-16]. It has been implicated in diverse neuropathologies, such as depression, schizophrenia and Alzheimer’s disease [17]. A novel mutation of the sigma-1R has been recently described in patients affected by juvenile ALS [18]. Sigma-1R agonists have demonstrated to promote protective effects reducing glutamate-mediated cell death [19,20] or modulating inflammatory reaction following stroke in rats [21]. Indeed, we have previously reported the therapeutic role of the selective sigma-1R agonist 2-(4-morpholi-nethyl)1-phenylcyclohexanecarboxylate (PRE-084) in SOD1^G93A^ ALS mice, that promoted spinal MN preservation and extended animal survival by controlling NMDA receptor calcium influx [22].

Resveratrol (RSV, 3,5,4’-trihydroxy-trans-stilbene) is a polyphenol naturally present in grapes and red wine that has been reported to exert neuroprotective effects on neurodegenerative disease models of Alzheimer’s disease [23] and Parkinson’s disease [24], and in traumatic [25] and ischemic injuries to the CNS [26]. Previous studies showed protective effects after RSV administration on in vitro ALS models [27,28]. We recently found that RSV administration to SOD1^G93A^ mice preserves spinal MN function and increases their survival. These beneficial effects were associated to activation of Sirtuin 1 (Sirt1) and AMP-activated protein kinase (AMPK) pathways, modulation of autophagy and an increase of mitochondrial biogenesis [29-32].

The pathophysiology of ALS is complex, with several processes contributing to MN death, including glutamate excitotoxicity, oxidative stress, protein misfolding, mitochondrial defects, impaired axonal transport and inflammation [33,34]. In this context, it is likely that combined therapies targeting several pathophysiological mechanisms could lead to stronger effects and better functional outcomes, potentially resulting in successful clinical translation. Thus, the main goal of this study is to assess if the combination of PRE-084 and RSV treatments may have synergistic effects in the SOD1^G93A^ ALS mice.

## Material and methods

### Transgenic mice and drug administration

Transgenic mice with the G93A human SOD1 mutation (B6SJL-Tg [SOD1-G93A]1Gur) were obtained from the Jackson Laboratory (Bar Harbor, ME, USA), and maintained at the Animal Service of the Universidad de Zaragoza. Hemizygotes B6SJL SOD1^G93A^ males were obtained by crossing with B6SJL females from the CBATEG (Bellaterra, Spain). The offspring was identified by PCR amplification of DNA extracted from the tail tissue. Mice were kept in standard conditions of temperature (22 ± 2°C) and a 12:12 light:dark cycle with access to food and water *ad libitum*. All experimental procedures were approved by the Ethics Committee of the Universitat Autònoma de Barcelona, where the animal experiments were performed.

Animals were evaluated at 8 weeks (prior to starting treatments) by rotarod and electrophysiological tests to obtain baseline values. Animals were distributed, according to their progenitors, weight and electrophysiological baseline values, in balanced experimental groups. A RSV-enriched diet [23] was given to groups of treated mice from 8 weeks of age. It has been previously demonstrated that RSV does not affect food intake of the animals [35], thereby assuming a normal food intake of 4 g/animal/day, then RSV was given at a daily dose of 160 mg/kg. PRE-084 (Tocris Bioscience, Ellisville, MO), a Sigma-1R agonist, was dissolved in saline and administered daily by single intraperitoneal injections at 0.25 mg/kg. The experimental groups included in the study are summarized in Table 1.

**Table 1:** Experimental groups included in the study

**Experimental group**	**Gender**	**n**
Wild type	Females	10
SOD1 untreated	Females	23
SOD1 + PRE	Females	18
SOD1 + RSV	Females	23
SOD1 + PRE + RSV	Females	21
Wild type	Males	10
SOD1 untreated	Males	10
SOD1 + PRE	Males	17
SOD1 + RSV	Males	12
SOD1 + PRE + RSV	Males	10

SOD1: SOD1^G93A^ mice; PRE: PRE-084 (0.25 mg/kg i.p.); RSV: resveratrol (160 mg/kg, via diet).

### Nerve conduction tests

Motor nerve conduction tests were performed at 8 weeks of age and then every two weeks until 16 weeks in all the animals used in the study. The sciatic nerve was stimulated percutaneously by means of single pulses of 0.02 ms duration (Grass S88) delivered through a pair of needle electrodes placed at the sciatic notch. The compound muscle action potential (CMAP, M wave) was recorded from the tibialis anterior (TA) and the plantar (interossei) muscles with microneedle electrodes [36,37]. All potentials were amplified and displayed on a digital oscilloscope (Tektronix 450S) at settings appropriate to measure the amplitude from baseline to the maximal negative peak. To ensure reproducibility, the recording needles were placed under microscope to secure the same placement on all animals guided by anatomical landmarks. During the tests, mouse body temperature was kept constant by means of a thermostated-controlled heating pad.

### Locomotion tests and clinical disease onset

The rotarod test was performed to evaluate motor coordination, strength and balance [38,39] in all the animals used in the study (Table 1). Mice were trained three times a week on the rod rotating at 14 rpm, and then tested from 8 to 16 weeks of age, with an arbitrary maximum time of maintenance in the rotating rod of 180 s. Clinical disease onset was defined as the first day when an animal was not able to complete the 180 seconds on the rotating rod.

### Survival

The mice were inspected daily until the standard end point or death. It was considered that animals reached the end point of the disease when they were unable to right themselves in 30s when placed on their side.

### Histology

At 16 weeks of age 4–5 mice of each group were transcardially perfused with 4% paraformaldehyde in PBS and the lumbar segment of the spinal cord was harvested, post-fixed for 24 h, and cryopreserved in 30% sucrose. Transverse 40 μm thick sections were serially cut with a cryotome (Leica) between L2-L5 segmental levels. For each segment, each section of a series of 10 was collected sequentially on separate gelatin-coated slides or free-floating in Olmos medium.

One slide of each animal was rehydrated for 1 min and stained for 2 h with an acidified solution of 3.1 mM cresyl violet. Then, the slides were washed in distilled water for 1 min, dehydrated and mounted with DPX (Fluka). MNs counts were made for neuronal soma present in the lateral part of lamina IX of the ventral horn in the spinal cord sections and following strict size and morphological criteria: only MNs with diameters larger than 20 μm, polygonal shape and prominent nucleoli were counted. The number of MNs present in both ventral horns was counted in four serial sections of each L4 and L5 segments [36,40], where motor nuclei innervating TA and plantar muscles are located [41].

Another series of sections was blocked with PBS-Triton-FBS and incubated overnight at 4°C with rabbit primary antibody against ionized calcium binding adaptor molecule 1 (Iba1, 1:1000, Wako). After several washes, sections were incubated for 1 hour at room temperature with Alexa 594-conjugated secondary antibody (1:200; Life Science). For co-localizations, spinal MNs were labeled with 500/525-Neurotrace fluorescent Nissl staining (1:200, Life Science). To quantify microglial immunoreactivity, microphotographs of the ventral horn grey matter were taken at × 400 and, after defining the threshold for background correction, the integrated density of Iba1 labeling was measured using ImageJ software. The integrated density represents the area above the threshold for the mean density minus the background.

### Protein extraction and western blot

For protein extraction, another subsets of mice (4–5 of each experimental group) were anesthetized and decapitated at 16 weeks of age. The lumbar spinal cord was removed and divided into quarters to isolate the ventral quadrants. One of them was prepared for protein extraction and homogenized in modified RIPA buffer (50 mM Tris–HCl pH 7.5, 1% Triton X-100, 0.5% sodium deoxycholate, 0.2% SDS, 100 mM NaCl, 1 mM EDTA) adding 10 μl/ml of Protease Inhibitor cocktail (Sigma) and PhosphoSTOP phosphatase inhibitor cocktail (Roche). After clearance, protein concentration was measured by Lowry assay (Bio-Rad Dc protein assay).

To perform western blots, 20–30 μg of protein of each sample were loaded in SDS-poliacrylamide gels. The transfer buffer was 25 mM trizma-base, 192 mM glycine, 20% (v/v) methanol, pH 8.4. The membranes were blocked with 5% BSA in TBS plus 0.1% Tween-20 for 1 hour, and then incubated with primary antibodies at 4°C overnight. The primary antibodies used were: anti-GAPDH (MAB374, Millipore, 1:20000), anti-Sirt1 (ab50517, Abcam, 1:1000), anti-p53 (#2524, Cell Signaling, 1:500), anti-acetyl p53 (L382) (06–758, Millipore, 1:500), anti-AMPK (#2532, Cell Signaling, 1:1000), anti-pAMPK (#2531, Cell Signaling, 1:1000), anti-NMDAR1 (AB9864, Millipore, 1:500) and anti-phospho NR1 (S896) (ABN88, Millipore, 1:500). Horseradish peroxidase–coupled secondary antibody (1:5000, Vector) incubation was performed for 60 min at room temperature. The membranes were visualized by enhanced chemiluminiscence method and the images were collected and analyzed with a Gene Genome apparatus using Gene Snap and Gene Tools software (Syngene, Cambridge, UK), respectively.

### Statistical analysis

Data are expressed as mean ± SEM. Electrophysiological and locomotion test results were statistically analyzed using repeated measurements and one-way ANOVA, applying Turkey post-hoc test when necessary. Histological data were analyzed using Mann–Whitney U test. Onset and survival data were analyzed using the Mantel-Cox test.

## Results

### Combined resveratrol and PRE-084 reduces locomotor impairments and delays clinical disease onset in SOD1^G93A^ mice

**Figure 1:**
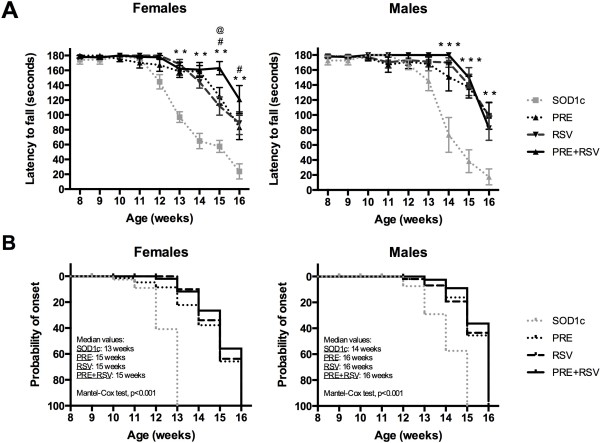
**PRE + RSV administration improves locomotor performance and delays disease onset in SOD1**^**G93A**^**mice. (A)** Rotarod test showed significant improvement in PRE + RSV treated animals compared to untreated littermates. Female but not male SOD1^G93A^ mice treated with PRE + RSV had better performance at advanced stages than RSV and PRE-084 alone treated mice. Values are expressed as mean ± SEM. **p < 0.01, ***p < 0.001 vs. untreated; #p < 0.05 vs. PRE-084 and @p < 0.05 vs. RSV treated SOD1^G93A^ mice. **(B)** Clinical onset analysis (Mantel-Cox test) revealed that PRE + RSV combination significantly delayed by 2 weeks the signs of disease onset both in female and male SOD1^G93A^ mice.

We assessed locomotor performance with the rotarod test [38,39]. Rotarod performance was significantly higher in both female and male PRE + RSV treated mice than in control SOD1^G93A^ mice (Figure 1). Furthermore, the combined treatment significantly improved motor outcome of female animals with respect to single RSV or PRE-084 mice at 15 and 16 weeks of age. However, in male mice PRE + RSV did not produce additional benefits compared to single treatments. Regarding clinical disease onset, PRE + RSV combination significantly delayed the first locomotor signs compared to untreated SOD1^G93A^ mice, but it not represented an improvement in comparison to single treated groups (Figure 1).

### Combined resveratrol and PRE-084 preserves spinal motoneuron function in SOD1^G93A^ mice

**Figure 2:**
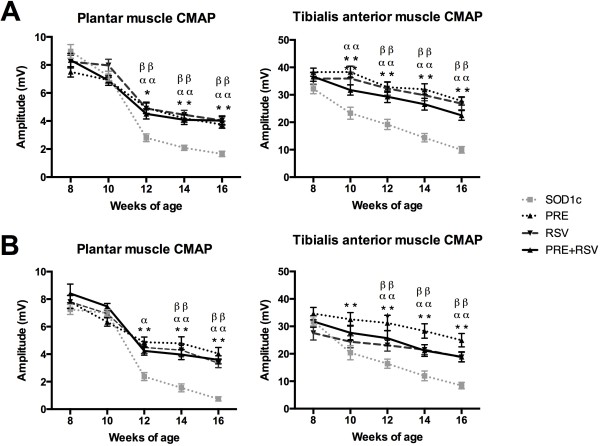
**PRE + RSV treatment preserves lower motoneuron function.** Electrophysiological tests performed in **(A)** female and **(B)** male SOD1^G93A^ mice revealed significant preservation of compound muscle action potentials (CMAP) amplitude in treated groups. Values are mean ± SEM. *p < 0.05, **p < 0.01 PRE-084 vs. untreated; α p < 0.05, αα p < 0.01 PRE + RSV vs. untreated; ββ p < 0.01 RSV vs. untreated.

Lower MN dysfunction is one the main clinical signs of ALS pathology both in animal models [36,41] and human patients [1]. We assessed the functional state of spinal MN by evaluating the amplitude of plantar and TA CMAPs. As previously reported [36] there was a progressive decline in CMAP amplitudes in both muscles along disease progression in SOD1^G93A^ untreated mice. The results revealed that PRE + RSV administration significantly preserves spinal MN function compared to SOD1^G93A^ untreated animals, although it did not promote a better outcome than separated RSV and PRE-084 treatments (Figure 2).

### Combined resveratrol and PRE-084 extends survival of SOD1^G93A^ mice

**Figure 3:**
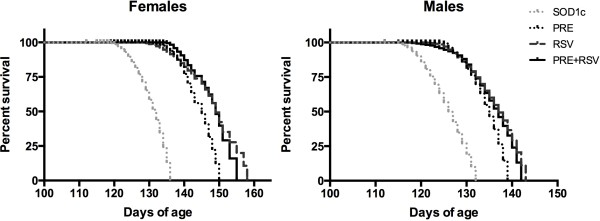
**PRE + RSV administration significantly extended SOD1**^**G93A**^**mice survival (Mantel-Cox test, p < 0.001)**. PRE + RSV treatment also increased animals survival compared to PRE-084-only treated group (Mantel-Cox test, p < 0.05).

RSV and PRE-084 combined administration from 8 weeks of age significantly extended both female (12.9%) and male (8.7%) lifespan compared to SOD1^G93A^ untreated mice (Mantel-Cox test, p < 0.001). The combined treatment also prolonged mice survival with respect to PRE-084 (Mantel-Cox test, p < 0.01) but not to RSV only treated SOD1^G93A^ mice (Figure 3).

### Combined resveratrol and PRE-084 administration reduces spinal motoneuron degeneration and reduces microglial reactivity in SOD1^G93A^ mice

**Figure 4:**
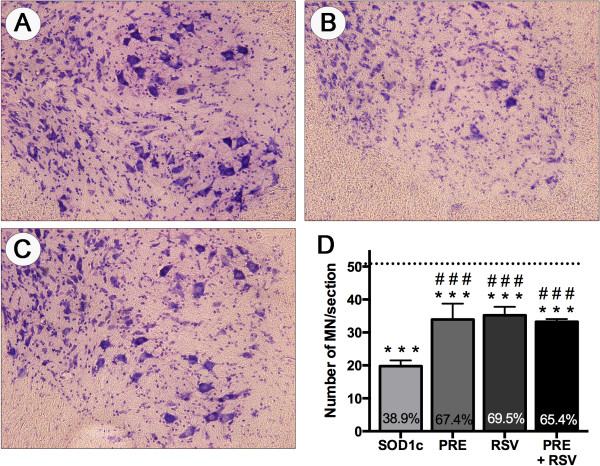
**PRE + RSV administration significantly preserves spinal motoneurons from degeneration.** Representative images of L4 spinal cord of **(A)** wild type, **(B)** SOD1^G93A^ untreated and **(C)** PRE + RSV SOD1^G93A^ treated mice. Scale bar, 500 μm. **(D)** L4-L5 spinal cord motoneuron counts revealed significant neuroprotection exerted by PRE + RSV co-administration compared to untreated SOD1^G93A^ but not to RSV-only or PRE-084-only treatments. Dashed line respresent the mean number of motoneurons per section of wild type mice. Values are mean ± SEM. ***p < 0.05 vs. wild type, ### p < 0.05 vs. untreated SOD1^G93A^ mice.

Spinal MN preservation was assessed by evaluating the number of Nissl stained MN cell bodies in the ventral horns of lumbar L4-L5 spinal segments in 16 weeks old SOD1^G93A^ mice. Figure 4 shows representative images of Nissl stained anterior horns to illustrate the differences between experimental groups. MN counts of female and male animals were pooled since there were no significant differences between genders. PRE-084 and RSV co-administration from 8 weeks of age significantly increased the number of preserved MNs in SOD1^G93A^ mice. PRE + RSV treated mice had 33.3 ± 1.74 MNs (65.4% vs. wild type) per section whereas SOD1^G93A^ untreated animals had 19.8 ± 0.79 (38.9% vs. wild type), almost a two-fold increase in surviving MNs. However, the combinatory treatment did not represent improvement in terms of neuronal preservation compared to single RSV or PRE-084 administered animals (Figure 4).

**Figure 5:**
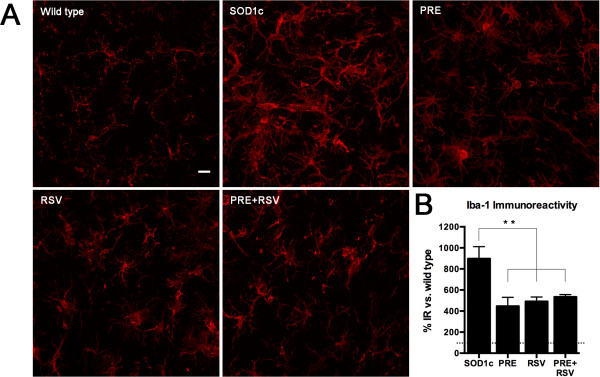
**PRE + RSV treatment significantly reduced Iba-1 immunoreactivity in L4-L5 spinal cord lamina IX. (A)** Representative microphotographs of L4-L5 spinal cord of wild type, untreated SOD1 (SOD1c), and PRE, RSV and PRE + RSV treated groups. **(B)** Percentage increase of Iba-1 immunoreactivity compared to wild type values. Values are mean ± SEM. **p < 0.01 vs. SOD1c.

We assessed the microglia reactivity as a measure of the inflammatory state of the lumbar spinal cord. PRE + RSV administration from 8 weeks of age significantly reduced the microglial immunoreactivity in lamina IX of L4-L5 spinal cord segments. However, similar improvement was observed with single PRE or RSV treatments (Figure 5).

### Combined resveratrol and PRE-084 increases Sirt1 and AMPK activation, and promotes specific PKC-depended phosphorylation of NMDA-NR1 subunits

We have previously shown that RSV treatment induced an increased expression and activation of Sirt1 and AMPK and a consequent modulation of autophagy and mitochondrial biogenesis [32]. On the other hand, PRE-084 administration leads to specific PKC-dependent NMDA-NR1 subunit phosphorylation that may protect MN from degeneration by reducing NMDA calcium currents and thus preventing excitotoxicity [14,22,42]. Therefore, we further analyzed whether RSV and PRE-084 co-administration similarly promotes the activation of the same downstream cellular pathways or there was any interference between drugs.

**Figure 6:**
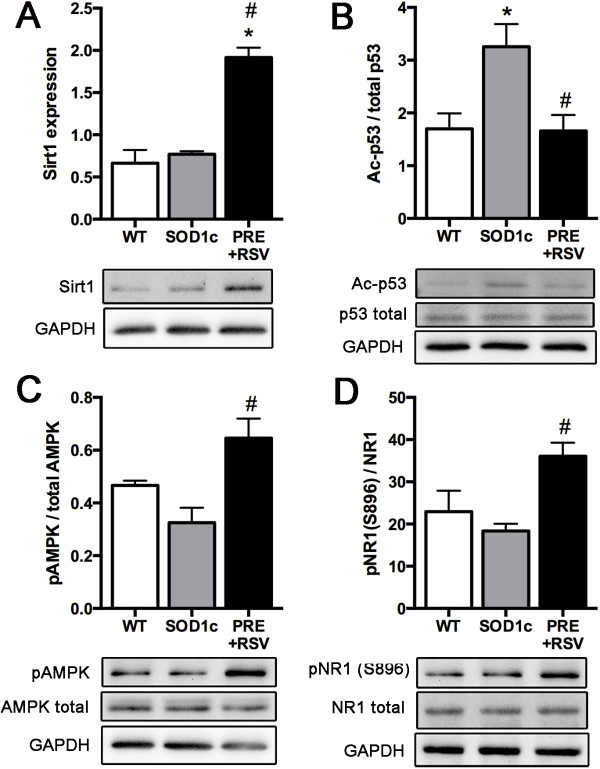
**Western blot analysis on the ventral lumbar spinal cord of animals co-administered with PRE + RSV. (A)** Increased sirtuin 1 expression in SOD1^G93A^ spinal motoneurons after PRE + RSV treatment. **(B)** Evaluation of Sirt1 activity by measuring the acetylation levels of p53 revealed significant deacetylation and thus, increased activity of Sirt1 in SOD1^G93A^ mice treated with PRE + RSV. **(C)** Enhanced AMPK activation after PRE + RSV treatment, evidenced by an increased pAMPK/AMPK ratio. **(D)** Increased Ser896 PKC-specific phosphorylation in NMDA-NR1subunits of animals treated with PRE + RSV. Values are mean ± SEM, *p < 0.05 vs. wild type, #p < 0.05 vs. untreated SOD1^G93A^ mice.

To assess RSV effects in the combinatory treated SOD1^G93A^ mice we first analyzed Sirt1 levels and activation in the ventral part of the lumbar spinal cord by western blot analysis. Results revealed a pronounced increase in Sirt1 expression after PRE + RSV co-administration (Figure 6A). To check whether this augmented expression was translated into an enhanced function we analyzed the acetylation state of one the most important Sirt1 targets, p53. We found a significant reduction of acetyl-p53 proportion indicating that Sirt1 was also over-activated (Figure 6B). Secondly, we assessed the activation of AMPK by evaluating active phospho-AMPK fraction. Results showed a marked increase in the pAMPK/AMPK ratio after PRE + RSV co-administration (Figure 6C). These changes were similar to those previously reported after treatment with RSV alone [32].

On the other hand, we evaluated the molecular effects of PRE-084 by analyzing the phosphorylation state of a PKC-specific serine (Ser896) in the NMDA-NR1 subunit. Western blot results showed increased Ser896 phosphorylation of the NMDA-NR1 subunit in the animals administered with PRE + RSV (Figure 6D).

## Discussion

The main goal of this study was to assess the effect of a novel therapeutic approach combining a Sigma-1R agonist, PRE-084, and RSV in the SOD1^G93A^ mouse model of ALS, since separate administration of the two compounds had resulted in significant improvement of disease progression and survival of these mice [22,32]. Our results indicate that co-administration of PRE-084 and RSV from 8 weeks of age significantly preserved spinal MNs function and reduced MN degeneration together with a reduction of microglial immunoreactivity in the lumbar spinal cord. This effect was accompanied by improvement in the locomotor performance and significant extension of the animals survival. Western blot analyses revealed that, as we previously described, PRE-084 induced PKC-specific phosphorylation of Ser896 of the NMDA-NR1 subunit, whereas RSV increased the expression and activation of Sirt1 and AMPK in the ventral part of the lumbar spinal cord of SOD1^G93A^ mice. Unfortunately, the combinatory therapy did not represent a clear improvement compared to RSV-only or PRE-084-only treated animals.

### Mechanisms of neuroprotection and possible overlapping effects

As we previously described, PRE-084 promotes potent neuroprotective effects to MNs both *in vitro* after excitotoxic insults [43], and *in vivo* after spinal root avulsion [11] and in the SOD1^G93A^ mouse model accompanied by a significant extension of animals survival [22]. Calcium dysregulation and excitotoxicity are two pathophysiological mechanisms contributing to ALS pathology [44,45]. In fact, spinal ALS-vulnerable MNs have an endogenous calcium buffering capacity 5–6 times lower than that found in ALS-resistant MNs, increasing their susceptibility to excitotoxic insults [46]. NMDA receptor plays an important role during excitotoxicity [45] and Sigma-1R agonists have been shown to suppress calcium influx to the cells by modulating NMDA receptor through PKC activation. Consistent with our previous observations that PRE-084 administration promotes PKC-specific NMDA-NR1 subunit phosphorylation [22], we have found that PRE + RSV combined treatment also increased Ser896 phosphorylation of the NDMA-NR1 subunit in the ventral part of the spinal cord. Interestingly, increased NMDA-NR1 phosphorylation was only present in the treated group, suggesting that the treatment is activating compensatory protective pathways rather than counteracting a pre-existent pathological event. Although it has been demonstrated that Sigma-1R physically interacts with NMDA-NR1 subunits [47], it has been also reported that Sigma-1R agonists can modulate ionic flow through calcium, sodium and potassium channels, thus modifying cell excitability properties [14,48,49]. In fact, recent findings by Mavlyutov et al. [50] indicate that the lack of Sigma-1R is detrimental in SOD1^G93A^ mice, probably because it acts by reducing the excitability of spinal MNs. Moreover, Sigma-1R is found associated to ER chaperones (such as BiP) and plays a role in clearance of misfolded proteins by the ERAD response [51,52]. Sigma-1R is enriched in the so-called mitochondrial-associated ER membrane (MAM) and its activation can also modulate mitochondrial metabolism [8,53].

It has been reported that RSV promotes protective effects both in neurodegenerative and traumatic injury models, including Alzheimer’s disease and accelerated aging [23,28,54], multiple sclerosis [55,56], Huntington’s disease [57,58], Parkinson’s disease [24,59], and reducing peripheral axonal degeneration [60] or promoting functional improvements after spinal cord injury [25]. We have recently found that RSV administration significantly delays clinical symptoms onset, improves spinal MNs function and survival, and extends SOD1^G93A^ mice lifespan. We also determined that the therapeutic effect was mediated by the increased expression and activation of both Sirt1 and AMPK, leading to normalization of the autophagic flux and enhanced mitochondrial biogenesis [32]. Although there is some controversy about the exact molecular mechanisms underlying RSV effect, it has been established that Sirt1 or AMPK activation are upstream of pathways that participate in several cellular processes, including inflammation [61-64], autophagy [32] and mitochondrial function [29,54]. Consistent with our previous findings, PRE + RSV treated animals also presented higher expression and activation of both Sirt1 and AMPK compared to SOD1^G93A^ untreated mice. As we previously commented regarding increased NMDA-NR1 phosphorylation, Sirt1 and AMPK overactivation was only present in the treated group, suggesting that the treatment activates protective pathways rather than counteracts a pre-existent pathological condition. This fact may increase the interest of these treatments since the potentiation of endogenous protective mechanisms can be translated to other non-SOD1 ALS situations.

Although the absence of summative effect in the PRE + RSV treated group may be due to an insufficient dose of any of the compounds, a possible overlapping in the pathways activated by both compounds may underlie the lack of synergy. As above mentioned, RSV protective effect is likely related to normalization of the autophagic flux and increased mitochondrial biogenesis [32]. Although PRE-084 main therapeutic effects have been considered associated to modulation of calcium influx and MNs excitability [14,48,49], it also participates on the response to misfolded protein accumulation [8,52] and the modulation of mitochondrial metabolism [53]. Considering this action, RSV effects on autophagy and mitochondrial biogenesis may mask those of PRE-084 administration thus explaining the lack of summative effect of the combined treatment. Alternatively, both compounds may exert opposite effects on some pathways. For example, it has been reported that Sigma-1R stabilize IRE1α and increase cell survival through transcriptional activity of X Box binding Protein 1 (XBP1) which in turn regulates genes responsible for protein folding and degradation during the Unfolded Protein Response (UPR) [51]. In contrast, RSV has been shown to suppress the transcriptional activity of XBP1 through Sirt1 [65], therefore promoting an opposite action that could contribute to the lack of synergistic effect of the combinatory treatment assayed.

Another explanation for the lack of summative effect of the combined treatment lies in the pathology state of the animals in the moment we began the drug administration. SOD1^G93A^ mice at 8 weeks of age present 25-30% loss of CMAP amplitude in proximal muscles (TA and gastrocnemius) [36,66], due to early neuromuscular junction retraction and motor axons degeneration [41]. Thus, although MN cell bodies in the anterior spinal cord are intact, up 30% of them are already under a degenerative process that may be irreversible. Since our treatment is focused on the preservation of remaining functional MNs, it is likely that we are acting on the still functioning population of MNs and thus, the maximum effect that can be reached would be limited.

### Does the SOD1^G93A^ mouse model present a limited therapeutic capacity?

An alterative explanation for the lack of summative effect after combining PRE-084 and RSV could be that the SOD1^G93A^ mouse model has a limit in terms of MN function and survival that cannot be overpassed by therapeutic interventions. To address this possibility, we made a review of successful preclinical trials using both single and combinatorial treatments in SOD1^G93A^ mice. Additional file 1: Table S1 shows a summary of the therapies performed and the percentage of increased survival compared to untreated mice. It is worth noting that no replication of the positive results was achieved for several of the drugs initially reported to provide efficacy when re-tested using a careful study design [67]. Up to our knowledge and without considering those works showing negative or null results, only 4 of 48 studies reported an extension of survival longer than 25% with just a few showing synergistic effect after combinatory approaches. Although deeper analyses must be performed, such observations may reflect an endogenous limitation for the therapeutical benefits that can be achieved using the SOD1^G93A^ mouse model.

## Conclusions

The main goal of the present work was to assess the therapeutic potential of a combinatory strategy using RSV and PRE-084 in the SOD1^G93A^ mouse model of ALS. Our results revealed that RSV and PRE-084 co-administration significantly improved MN functional preservation and neuroprotection, accompanied by an improvement of the locomotor performance and survival extension. However, this effect was not comparatively better than that achieved by administration of RSV alone.

## Competing interest

The authors declare that they have not competing interests.

## Author’s contribution

RM designed the study, performed functional tests and histological studies, collected and analyzed the data, made the figures and prepared the manuscript; JdV performed molecular biology analyses; MM performed the locomotion evaluation; RO breaded and genotyped the animals; MP contributed to set the treatment diet and to prepare the manuscript; XN conceived and designed the study, supervised data analysis and prepared the manuscript. All authors read and approved the final manuscript.

## Supplementary Material

Additional file 1: Table S1 Summary of recent therapeutic trials performed in the SOD1^G93A^ mouse model. Click here for file
