# Supplementary material for: Lack of synergistic effect of resveratrol and sigma-1 receptor agonist (PRE-084) in SOD1G93A ALS mice: overlapping effects or limited therapeutic opportunity?
Source: Orphanet J Rare Dis. 2014 May 21;9:78. doi: 10.1186/1750-1172-9-78 (PMC4035830; doi:10.1186/1750-1172-9-78)
Supplement: Additional file 1: Table S1 — Summary of recent therapeutic trials performed in the SOD1G93A mouse model. [file 1750-1172-9-78-S1.docx]

**Additional file 1: Table 1**. Summary of recent therapeutic trials performed in the SOD1^G93A^ mouse model. When dose-dependent or gender effects were described, the greatest improvements were selected.

| **Compound** | **Treatment onset (days)** | **Route** | **Survival extension** | **Ref** |
| --- | --- | --- | --- | --- |
| *Oxidative stress* |  |  |  |  |
| M30 | Pre-symptomatic (70) | Oral | 6% | [1] |
| Metallothionein-III | Onset (90) | I.M | 10% | [2] |
| VK-28 | Pre-symptomatic (60) | I.P | 10% | [3] |
| Dispocynin | Post-symptomatic (100) | Oral | 8% | [4] |
| Apocynin | Post-symptomatic (100) | Oral | 2.3% | [4] |
| *Mitochondrial dysfunction* |  |  |  |  |
| Uridine | Pre-symptomatic (42) | I.P | 17% | [5] |
| Dichloroacetate | Pre-symptomatic (70) | Oral | 9% | [6] |
| *Excitotoxicity* |  |  |  |  |
| Methionine sulfoximine | Pre-symptomatic (50) | I.P | 8% | [7] |
| *Protein missfolding* |  |  |  |  |
| Immunization against mSOD1 | Pre-symptomatic (65) | I.VT | 6.5% | [8] |
| Ariloxanyl | Pre-symptomatic (42) | I.P | 16% | [9] |
| Cyclohexane | Pre-symptomatic (42) | I.P | 18% | [10] |
| *Trophic support* |  |  |  |  |
| Lead | Early pre-symptomatic (21) |  | 10% | [11] |
| VEGF (AVV4-mediated) | Onset (80) | I.VT | 16% | [12] |
| IGF-1 (AVV4-mediated) | Onset (80) | I.VT | 10% | [12] |
| BDNF-TTC | Pre-symptomatic (56) | I.M | 14% | [13] |
| *Non-cell autonomous/inflammation* |  |  |  |  |
| Revlimid | Onset (90) | Oral | 12% | [14] |
| GCSF (AAV1/2-mediated) | Pre-symptomatic (70) | I.TH | 10% | [15] |
| Monoclonal antibodies anti-CD40L | Pre-symptomatic (50) | I.P | 7% | [16] |
| Minocicline | Early Pre-symptomatic (35) | I.P | 8.8% | [17] |
| Pegfilgastrim | Onset (90) | S.C | 6% | [18] |
| Anti-Ly6C monoclonal antibodies | Onset (80) | I.P | 16% | [19] |
| Caffeic acid | Onset (90) | Oral | 7% | [20] |
| *Other* |  |  |  |  |
| L-arginine | Early pre-symptomatic (30) | Oral | 20% | [21] |
|  | Pre-symptomatic (70) | Oral | 9% | [21] |
| Activated protein C | Post-onset (97) | I.P | 22% | [22] |
| SUN N8075 | Pre-symptomatic (70) | S.C | 11% | [23] |
| TTC | Pre-symptomatic (56) | I.M | 12% | [24] |
| Diallyl trisulfide | Onset (90) | Oral | 5% | [25] |
| CDDO ethylamide | Early pre-symptomatic (30) | Oral | 16% | [26] |
|  | Onset (80) | Oral | 13% | [26] |
| CDDO trifluoroethylamide | Early pre-symptomatic (30) | Oral | 14% | [26] |
|  | Onset (80) | Oral | 13% | [26] |
| Trichostatin A | Onset (90) | I.P | 7% | [27] |
| AGS-499 | Pre-symptomatic (70) | S.C | 16% | [28] |
| PRE-084 | Pre-symptomatic (56) | I.P. | 16% | [29] |
| Dihydrotestosterone | Pre-symptomatic (75) | S.C | 5% | [30] |
| Bromocriptine methylase | Onset (90) | I.P | 2% | [31] |
| CPN-9 | Onset (90) | I.G | 16% | [32] |
| *Multiple pathways* |  |  |  |  |
| Lithium chloride | Pre-symptomatic (67) | I.P | 8% | [33] |
| Resveratrol | Pre-symptomatic (56) | Oral | 10.5% | [34] |
| *Combinatorial strategies* |  |  |  |  |
| VEGF+IGF-1 (AAV4-mediated) | Onset (80) | I.VT | 8% | [12] |
| IGF-1 (AVV2-mediated) + exercise | Early pre-symptomatic (90-40, respectively) | I.M | **69.7%** | [35] |
|  | Onset (90 in both cases) | I.M | **31%** | [35] |
| Minocycline + Creatine | Early pre-symptomatic (28-21, respectively) | Oral; I.P | 25% | [36] |
| NEU2000 + Lithium chloride | Pre-symptomatic (56) | Oral | 21.5% | [37] |
| PBA + AEOL 10150 | Onset (90) | I.P | 19% | [38] |
| Creatine + calecoxib | Early pre-symptomatic (30) | Oral | **28.5%** | [39] |
| Creatine + rafecoxib | Early pre-symptomatic (30) | Oral | **30.5%** | [39] |

Bold letter label those strategies that resulted in more than a 25% of increased survival. When the clinical disease onset was not specified, it was assumed as 90 days of age. S.C=subcutaneous; I.P=intraperitoneal; I.M=intramuscular; I.VT=intraventricular; I.TH=intrathecal; I.G=intragastric.

**References**

1. Kupershmidt L, Weinreb O, Amit T, Mandel S, Carrì MT, Youdim MBH. **Neuroprotective and neuritogenic activities of novel multimodal iron-chelating drugs in motor-neuron-like NSC-34 cells and transgenic mouse model of amyotrophic lateral sclerosis**. *FASEB J* 2009;**23**:3766–79.

2. Hashimoto K, Hayashi Y, Watabe K, Inuzuka T, Hozumi I. **Metallothionein-III prevents neuronal death and prolongs life span in amyotrophic lateral sclerosis model mice**. *Neuroscience* 2011;**189**:293–8.

3. Wang Q, Zhang X, Chen S, Zhang X, Zhang S, Youdium M, Le W. **Prevention of motor neuron degeneration by novel iron chelators in SOD1(G93A) transgenic mice of amyotrophic lateral sclerosis**. *Neurodegenerative Dis* 2011;**8**:310–21.

4. Trumbull KA, McAllister D, Gandelman MM, Fung WY, Lew T, Brennan L, Lopez N, Morré J, Kalyanaraman B, Beckman JS. **Diapocynin and apocynin administration fails to significantly extend survival in G93A SOD1 ALS mice**. *Neurobiol Dis* 2012;**45**:137–44.

5. Amante DJ, Kim J, Carreiro ST, Cooper AC, Jones SW, Li T, Moody JP, Edgerly CK, Bordiuk OL, Cormier K, Smith K, Ferrante RJ, Rusche KJ. **Uridine ameliorates the pathological phenotype in transgenic G93A-ALS mice**. *Amyotroph Lateral Scler* 2010;**11**:520–30.

6. Miquel E, Cassina A, Martinez-Palma L, Bolatto C, Trias E, Gandelman M, Radi R, Barbeito LH, Cassina P. **Modulation of astrocytic mitochondrial function by dichloroacetate improves survival and motor performance in inherited amyotrophic lateral sclerosis**. *PLoS ONE* 2012;**7**:e34776.

7. Ghoddoussi F, Galloway MP, Jambekar A, Bame M, Needleman R, Brusilow WSA. **Methionine sulfoximine, an inhibitor of glutamine synthetase, lowers brain glutamine and glutamate in a mouse model of ALS**. *J Neurol Sci* 2010;**290**:41–7.

8. Gros-Louis F, Soucy G, Larivière R, Julien JP. **Intracerebroventricular infusion of monoclonal antibody or its derived Fab fragment against misfolded forms of SOD1 mutant delays mortality in a mouse model of ALS**. *J Neurochem* 2010;**113**:1188–99.

9. Chen T, Benmohamed R, Kim J, Smith K, Amante D, Morimoto RI, Kirsch DR, Ferrante RJ, Silverman RB. **ADME-guided design and synthesis of aryloxanyl pyrazolone derivatives to block mutant superoxide dismutase 1 (SOD1) cytotoxicity and protein aggregation: potential application for the treatment of amyotrophic lateral sclerosis**. *J Med Chem* 2012;55:515–27.

10. Zhang Y, Benmohamed R, Zhang W, Kim J, Edgerly CK, Zhu Y, Morimoto RI, Ferrante RJ, Kirsch DR, Silverman RB. **Chiral cyclohexane 1,3-diones as inhibitors of mutant SOD1-dependent protein aggregation for the treatment of ALS**. *ACS Med Chem Lett* 2012;**3**:584–7.

11. Barbeito AG, Martinez-Palma L, Vargas MR, Pehar M, Mañay N, Beckman JS, Barbeito LH, Cassina P. **Lead exposure stimulates VEGF expression in the spinal cord and extends survival in a mouse model of ALS**. *Neurobiol Dis* 2010;**37**:574–80.

12. Dodge JC, Treleaven CM, Fidler JA, Hester M, Haidet A, Handy C, Rao M, Eagle A, Matthews JC, Taksir TV, Cheng SH, Shihabuddin LS, Kaspar BK. **AAV4-mediated expression of IGF-1 and VEGF within cellular components of the ventricular system improves survival outcome in familial ALS mice**. *Mol Ther* 2010;**18**:2075–84.

13. Calvo AC, Moreno-Igoa M, Mancuso R, Manzano R, Oliván S, Muñoz MJ, Penas C, Zaragoza P, Navarro X, Osta R. **Lack of a synergistic effect of a non-viral ALS gene therapy based on BDNF and a TTC fusion molecule**. Orphanet J Rare Dis. 2011;6:10.

14. Neymotin A, Petri S, Calingasan NY, Wille E, Schafer P, Stewart C, Hansley K, Beal MF, Kiaei M. **Lenalidomide (Revlimid) administration at symptom onset is neuroprotective in a mouse model of amyotrophic lateral sclerosis**. *Exp Neurol* 2009;**220**:191–7.

15. Henriques A, Pitzer C, Dittgen T, Klugmann M, Dupuis L, Schneider A. **CNS-targeted viral delivery of G-CSF in an animal model for ALS: improved efficacy and preservation of the neuromuscular unit**. *Mol Ther* 2011;**19**:284–92.

16. Lincecum JM, Vieira FG, Wang MZ, Thompson K, De Zutter GS, Kidd J, Moreno A, Sanchez R, Carrion IJ, Levine BA, Al Nakhala BM, Sullivan SM, Gill A, Perrin S. **From transcriptome analysis to therapeutic anti-CD40L treatment in the SOD1 model of amyotrophic lateral sclerosis**. *Nat Genet* 2010;**42**:392–9.

17. Zhu S, Stavrovskaya IG, Drozda M, Kim BYS, Ona V, Li M, Sarang S, Liu AS, Hartley DM, Wu DC, Gullans S, Ferrante RJ, Przedborski S, Kristal BS, Friedlander RM. **Minocycline inhibits cytochrome c release and delays progression of amyotrophic lateral sclerosis in mice**. *Nature* 2002;**417**:74–8.

18. Pollari E, Savchenko E, Jaronen M, Kanninen K, Malm T, Wojciechowski S, Ahtoniemi T, Goldstein G, Giniatullina R, Giniatullin R, Koistinaho J, Magga J. **Granulocyte colony stimulating factor attenuates inflammation in a mouse model of amyotrophic lateral sclerosis**. J Neuroinflamm 2011;**8**:74.

19. Butovsky O, Siddiqui S, Gabriely G, Lanser AJ, Dake B, Murugaiyan G, Doykan CE, Wu PM, Gali RR Iyer LK, Lawson R, Berry J, Krichevsky AM, Cudkowicz E, Weiner HL. **Modulating inflammatory monocytes with a unique microRNA gene signature ameliorates murine ALS**. *J Clin Invest* 2012;**122**:3063–87.

20. Fontanilla CV, Wei X, Zhao L, Johnstone B, Pascuzzi RM, Farlow MR, Du Y. **Caffeic acid phenethyl ester extends survival of a mouse model of amyotrophic lateral sclerosis**. *Neuroscience* 2012;**205**:185–93.

21. Lee J, Ryu H, Kowall NW. **Motor neuronal protection by L-arginine prolongs survival of mutant SOD1 (G93A) ALS mice**. *Biochem Biophys Res Commun* 2009;**384**:524–9.

22. Zhong Z, Ilieva H, Hallagan L, Bell R, Singh I, Paquette N, Thiyagarajan M, Deane R, Fernandez JA, Lane S, Zlokovic AB, Liu T, Griffin JH, Chow N, Castellino FJ, Stojanovic K, Cleveland DW, Zlokovic BV. **Activated protein C therapy slows ALS-like disease in mice by transcriptionally inhibiting SOD1 in motor neurons and microglia cells**. *J Clin Invest* 2009;**119**:3437–49.

23. Shimazawa M, Tanaka H, Ito Y, Morimoto N, Tsuruma K, Kadokura M, Tamura S, Inoue T, Yamada M, Takahashi H, Warita H, Aoki M, Hara H. **An inducer of VGF protects cells against ER stress-induced cell death and prolongs survival in the mutant SOD1 animal models of familial ALS**. *PLoS ONE* 2010;**5**:e15307.

24. Moreno-Igoa M, Calvo AC, Penas C, Manzano R, Oliván S, Muñoz MJ, Mancuso R, Zaragoza P, Aguilera J, Navarro X, Osta R. **Fragment C of tetanus toxin, more than a carrier. Novel perspectives in non-viral ALS gene therapy**. *J Mol Med* 2010;**88**:297–308.

25. Guo Y, Zhang K, Wang Q, Li Z, Yin Y, Xu Q, Duan W, Li C. **Neuroprotective effects of diallyl trisulfide in SOD1-G93A transgenic mouse model of amyotrophic lateral sclerosis**. *Brain Res* 2011;**1374**:110–5.

26. Neymotin A, Calingasan NY, Wille E, Naseri N, Petri S, Damiano M, Liby KT, Risingsong R, Sporn M, Beal MF, Kiaei M. **Neuroprotective effect of Nrf2/ARE activators, CDDO ethylamide and CDDO trifluoroethylamide, in a mouse model of amyotrophic lateral sclerosis**. *Free Rad Biol Med* 2011;**51**:88–96.

27. Yoo Y-E, Ko C-P. **Treatment with trichostatin A initiated after disease onset delays disease progression and increases survival in a mouse model of amyotrophic lateral sclerosis**. *Exp Neurol* 2011;**231**:147–59.

28. Eitan E, Tichon A, Gazit A, Gitler D, Slavin S, Priel E. **Novel telomerase-increasing compound in mouse brain delays the onset of amyotrophic lateral sclerosis**. *EMBO Mol Med* 2012;**4**:313–29.

29. Mancuso R, Oliván S, Rando A, Casas C, Osta R, Navarro X. **Sigma-1R Agonist Improves Motor Function and Motoneuron Survival in ALS Mice**. *Neurotherapeutics* 2012;**9**:814–26.

30. Yoo Y-E, Ko C-P. **Dihydrotestosterone ameliorates degeneration in muscle, axons and motoneurons and improves motor function in amyotrophic lateral sclerosis model mice**. *PLoS ONE* 2012;**7**:e37258.

31. Tanaka K, Kanno T, Yanagisawa Y, Yasutake K, Hadano S, Yoshii F, Ikeda J. **Bromocriptine methylate suppresses glial inflammation and moderates disease progression in a mouse model of amyotrophic lateral sclerosis**. *Exp Neurol* 2011;**232**:41–52.

32. Kanno T, Tanaka K, Yanagisawa Y, Yasutake K, Hadano S, Yoshii F, Hirayama N, Ikeda J. **A novel small molecule, N-(4-(2-pyridyl)(1,3-thiazol-2-yl))-2-(2,4,6-trimethylphenoxy) acetamide, selectively protects against oxidative stress-induced cell death by activating the Nrf2-ARE pathway: therapeutic implications for ALS**. *Free Rad Biol Med* 2012;**53**:2028–42.

33. Ferrucci M, Spalloni A, Bartalucci A, Cantafora E, Fulceri F, Nutini M, Longone P, Paparelli A, Fornai F. **A systematic study of brainstem motor nuclei in a mouse model of ALS, the effects of lithium**. *Neurobiol Dis* 2010;**37**:370–83.

34. Mancuso R, del Valle J, Modol L, Martinez A, Granado-Serrano AB, Ramirez-Núñez O, Pallás M, Portero-Otin M, Osta R, Navarro X. **Resveratrol improves motoneuron function and extends survival in SOD1^G93A^ ALS mice**. *Neutotherapeutics* 2014; in press.

35. Kaspar BK, Frost LM, Christian L, Umapathi P, Gage FH. **Synergy of insulin-like growth factor-1 and exercise in amyotrophic lateral sclerosis**. *Ann Neurol* 2005;**57**:649–55.

36. Zhang W, Narayanan M, Friedlander RM. **Additive neuroprotective effects of minocycline with creatine in a mouse model of ALS**. *Ann Neurol* 2003;**53**:267–70.

37. Shin JH, Cho SI, Lim HR, Lee JK, Lee YA, Noh JS, Joo IS, Kim KW, Gwag BJ. **Concurrent administration of Neu2000 and lithium produces marked improvement of motor neuron survival, motor function, and mortality in a mouse model of amyotrophic lateral sclerosis**. Mol Pharmacol. 2007 Jan 12;71(4):965–75.

38. Petri S, Kiaei M, Kipiani K, Chen J, Calingasan NY, Crow JP, Beal MF. **Additive neuroprotective effects of a histone deacetylase inhibitor and a catalytic antioxidant in a transgenic mouse model of amyotrophic lateral sclerosis**. *Neurobiol Dis* 2006;**22**:40–9.

39. Klivenyi P, Kiaei M, Gardian G, Calingasan NY, Beal MF. **Additive neuroprotective effects of creatine and cyclooxygenase 2 inhibitors in a transgenic mouse model of amyotrophic lateral sclerosis**. *J Neurochem* 2003;**88**:576–82.
